# Exploring green proteins from pumpkin leaf biomass: assessing their potential as a novel alternative protein source and functional alterations via pH-Shift treatment

**DOI:** 10.1186/s40643-025-00945-x

**Published:** 2025-09-23

**Authors:** Marija Korićanac, Jelena Mijalković, Predrag Petrović, Neda Pavlović, Zorica Knežević-Jugović

**Affiliations:** 1 Faculty of Technology and Metallurgy, Department of Biochemical Engineering and Biotechnology, University of Belgrade, Karnegijeva 4, Belgrade, 11000 Serbia; 2 Innovation Centre of the Faculty of Technology and Metallurgy Ltd, Karnegijeva 4, Belgrade, 11000 Serbia

**Keywords:** Green proteins, Pumpkin leaf biomass, Green biomass protein biorefinery, Protein modification, PH-shift treatment, Alternative protein source, Sustainability, Circular economy

## Abstract

As sustainability continues to be a major global focus, the use of agro-industrial waste as a renewable resource presents a promising solution. In this study, the potential of pumpkin leaf biomass, a type of agro-industrial waste, will be explored as an alternative protein source using thermal coagulation. A key challenge is the accumulation of green proteins, a protein-rich byproduct. This waste fraction, which has been insufficiently investigated due to its limited functional properties, is the main focus of this research, aimed at enabling complete biomass utilization. The novelty of this research lies in the application of combined pH-shift and controlled heat treatment to improve the solubility of green proteins isolated from pumpkin leaf biomass – an underexplored protein fraction derived from agro-industrial waste. The obtained crude green protein powder, with a yield of 47.95 g/kg of leaf dry biomass, exhibited a satisfactory composition, containing 53.58% protein, a high-quality amino acid profile, and notable antioxidant properties. On the other hand, it exhibited low solubility, below 25%, across the pH range of 2–10, with the isoelectric point at pH 4.4. Following the application of an optimized pH-shift and heat treatment, green proteins solubility increased significantly, reaching 89.74% at pH 8, nearly 4.5 times higher than before treatment, showing low solubility only at the isoelectric point and excellent stability maintained in salt concentrations up to 1 M NaCl. A notable decrease in particle size was observed, from 1883 nm to 192 nm, leading to a more uniform particle size distribution. SDS-PAGE and FTIR deconvolution analyses indicated structural changes, possibly involving disulfide bond formation. The promising findings of this research, not only ensure complete biomass utilization, but also highlight the need for further investigation regarding the potential of green modified proteins, as the observed improvements open up numerous biotechnological applications. One potential direction is green protein-stabilized emulsions, supported by a significant increase in alpha-helix content associated with enhanced functional properties, which offers new possibilities for future research on stable and functional emulsions.

**Supplementary Information:**

The online version contains supplementary material available at 10.1186/s40643-025-00945-x.

## Graphical Abstract

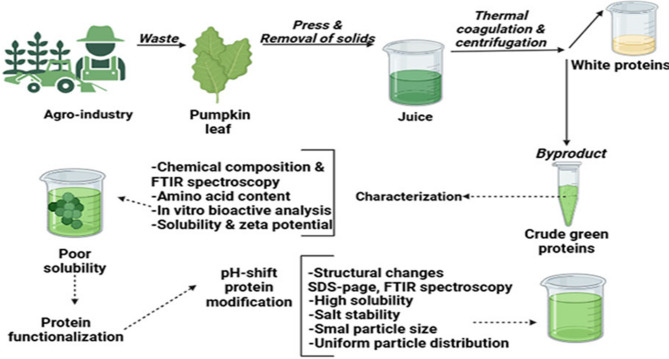

## Introduction

Searching for alternative protein sources has become urgent due to the insufficiency of traditional food sources and global challenges amplified by climate change. With sustainability in focus, plant proteins, especially those from agro-industrial waste, offer an attractive solution. While often used for animal feed or burned, this waste holds significant potential. It contains valuable nutrients that, when processed, can contribute to a circular economy, reducing waste and promoting resource reuse (Hadidi et al. 2024).

In this context, proteins from leaf biomass have garnered significant attention due to their uniform composition and consistent amino acid content across different crops (Fiorentini and Galoppini 1983). The protein content of leaves, as a potential food source, is quite satisfactory, with an average content ranging from 10.8% to 35.7% of dry matter, highlighting their potential as an alternative protein source (Balfany et al. 2023). In the search for alternative plant-based proteins, an interesting approach is to utilize green biomass, such as pumpkin leaves, particularly from hulless-seeded pumpkin or oil pumpkin (lat. *Cucurbita pepo* var. *oleifera*), which are not typically intended for consumption and therefore remain as waste material in the fields. Nevertheless, it was found that pumpkin leaves have quite high protein content, near to the upper limit of the average protein content in leaves, giving them significant advantage (Balfany et al. 2023; Mijalković et al. 2024). Furthermore, the cultivation of pumpkin leaves does not require special conditions, as they generated as a byproduct (waste green biomass) of pumpkin fruit and seed oil production. Thus, pumpkin leaves offer significant potential as a protein source, making them even more intriguing due to their limited exploration in terms of utility.

Approximately 80% of leaf proteins are located within the chloroplasts, evenly distributed between the soluble phase (stroma) and the lamellar membrane system (thylakoids), which are known as the white and green protein fractions, respectively (Fiorentini and Galoppini 1983). The thylakoid system contains pigment-protein complexes, along with their light-harvesting proteins and cofactors, which are responsible for electron transport during photosynthesis (Rascio 2013). The photosynthetic electron transport chain consists of four supramolecular complexes, often referred to as green proteins: three of these complexes are interconnected by mobile electron carriers (photosystem I, photosystem II and cyt b-f), and the fourth complex, ATP synthase, facilitates energy conversion. Each of these protein systems has an intricate structure composed of numerous subunits (Anderson 1982). These organized structures are embedded within a lipid bilayer of glycerolipids which are directly and indirectly involved in photosynthesis (Kobayashi 2016).

Due to the complex structure of green proteins as membrane proteins, their poor functional properties, green color, and grassy flavor, the use of organic solvents and detergents for their extraction is often necessary (Tenorio et al. 2016, 2017a). Thus, leaf protein isolation techniques are designed to target the white protein fraction, with thermal coagulation being the most commonly used method, as green proteins are thermally unstable. This instability facilitates the efficient separation of these two fractions. As a result, approximately 50% of the total leaf proteins remain as a byproduct (green protein), which is typically used for animal feed (Balfany et al. 2023; Pandey and Srivastava 1993).

Nevertheless, interest in green proteins is steadily increasing, despite their limitations and green color. Several studies have investigated thylakoid-stabilized emulsions, although further research is needed (Rayner et al. 2011; Tenorio et al. 2017b). Additionally, the poor functional properties of these proteins can be addressed through well-established protein extraction methods, fractionation techniques, and modifications (Sim et al. 2021). The main objective is to improve protein solubility, as it indirectly affects other properties such as emulsification and foaming (Grossmann and McClements 2023). To our knowledge, however, there has been limited research focused on enhancing the solubility of green proteins. Recently, the pH-shift method has gained attention as a relatively simple and effective approach. The principle behind this method is exposing proteins to an extremely low or high pH environment, followed by readjustment to a neutral or slightly alkaline one, which induces conformational changes and enhances protein hydrophilicity (Jiang et al. 2018). Moreover, combining an alkaline environment with heat has shown to produce exceptional results (Wang et al. 2018).

The main objective of this study is to examine the potential of pumpkin leaf agro-industrial waste as an alternative protein source, with its complete utilization. Leaf protein isolation will be performed through thermal coagulation, with a focus on the byproduct, green protein, due to its limited research. As this is a preliminary investigation, the aim is to explore crude green proteins with-out further purification. The primary goal is to evaluate the potential of pumpkin green protein as an alternative protein source, focusing on its composition, nutritional value, and solubility as a fundamental protein property. Additionally, the study addressed the functionalization of green proteins by improving their solubility using the pH-shift method and examining the synergistic effect of its combination with controlled heat treatment, applied to green proteins for the first time.

## Materials and methods

### Pumpkin leaf biomass preparation and chemical reagents

Pumpkin (lat. *Cucurbita pepo* var. *oleifera*) leaves, whose harvest was carried out in 2023 and 2024, were kindly provided by JS&O d.o.o Novo Miloševo, Novo Miloševo, Serbia. The leaves were cleaned of dirt and then stored in a deep freezer at − 80 °C until use.

The chemical reagents needed for the protein content spectrometric analysis, including bovine serum albumin, Folin and Ciocalteu’s phenol reagent, have been obtained from Sigma Aldrich Co. (St. Louis, MO, USA). The Megazyme total dietary fiber kit and remaining chemical reagents that were utilized were all analytical grade. Using a Milli-Q purification system (Merck Millipore Advantage A10, Darmstadt, Germany), deionized water (18.2 M MΩ.cm) was produced for sample solubilization.

### Protein isolation from pumpkin leaf biomass

**Scheme 1:**
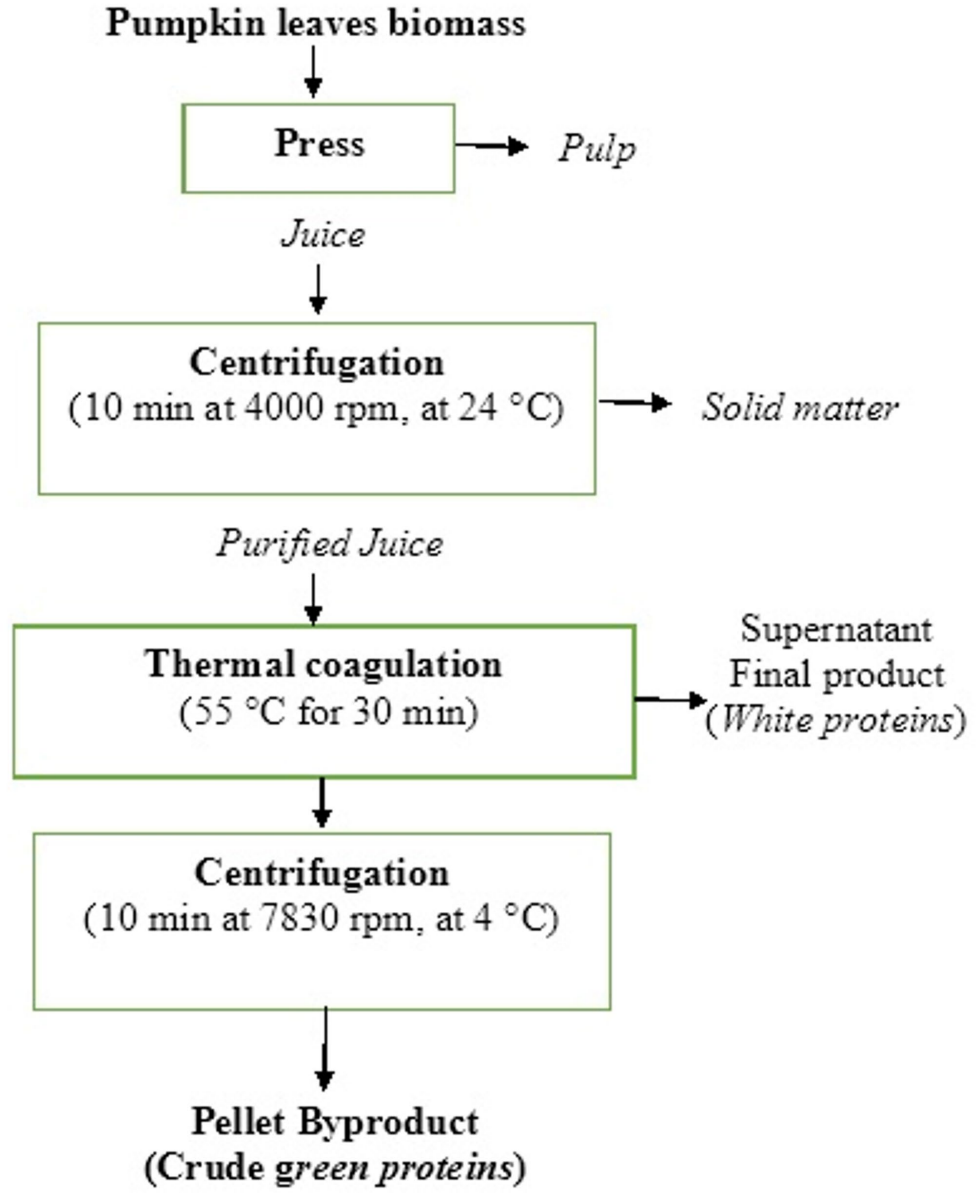
Schematic overview of the crude green protein’s isolation process

The isolation of pumpkin leaf proteins was carried out using the common method for white protein isolation, which involves pressing pumpkin leaves to obtain green juice, followed by thermal precipitation. After centrifugation, the white proteins remain in the supernatant, while green protein fraction precipitates and is typically considered a byproduct. The process was carried out as follows: The frozen pumpkin leaves were incubated at room temperature until they were thawed but still cool. Afterwards, the leaves were mechanically crushed using a twin screw-press (Angel juicer model 8500, Angel Juicer Co. LTD, South Korea), resulting in two defined fractions: pulp and green juice. The juice was then centrifuged (ultracentrifuge Optima XPN-100-IVD model, Beckman Coulter, Inc., California, USA) at 4000 rpm for 10 min at room temperature, in order to separate the remaining solid matter. The resulting supernatant was thermally coagulated for 30 min at 55 °C, followed by centrifugation for 10 min at 7830 rpm at 4 °C after cooling. The obtained crude green pellet (byproduct) was collected, resuspended in water and lyophilized (Beta 1–8 LSC basic Freeze Dryer, Martin Christ GmbH, Osterode am Harz, Germany) by following conditions: primary drying at − 40 °C for 23 h, and then final drying at − 60 °C for 1 h. The following scheme illustrates the crude green protein isolation process outlined above (Scheme 1):

#### Determining protein distribution and process efficiency

In order to determine the protein mass balance throughout the isolation process, proteins were quantified in both the supernatants and pellets at each isolation step. These samples were collected separately from the starting material (pumpkin leaves) to the final product, the crude green protein fraction. Furthermore, each fraction was lyophilized, after which the moisture content was measured using a moisture analyzer (Kern MLS-A, Balingen, Germany). Protein content was then quantified using the modified Lowry method (Hartree 1972). Then, for each step, the obtained protein concentration was expressed as a percentage relative to the total protein content of the pumpkin leaves (100%).

For the sake of accuracy, in addition to the modified Lowry method, protein concentration in pumpkin leaves and the green protein fraction was also determined using the Dumas method (AOAC 2023) which is based on the nitrogen content determination. For the final calculation, the total nitrogen was multiplied by a protein factor of 6.25. Furthermore, process efficiency was expressed as the mass of green proteins obtained from 1 kg of dry leaf biomass.

### Composition analysis

#### Determination of protein content by modified lowery method

The protein content was determined according to the modified Lowry method. Before analysis, the crude green protein powder was dissolved in water to obtain a concentration of 10 mg/mL. It was then stirred on a magnetic stirrer (IKA Magnetic Stirrers C-MAG HS 7 with ETS-D6, IKA-Werke GmbH & Co. KG, Germany) at 250 rpm until fully hydrated. Afterwards, an aliquot of the sample was prepared as described (Hartree 1972). The analysis was performed in duplicate and measurements were made in triplicate. Protein concentration was determined using BSA as the standard protein.

#### Chemical composition

For quantitative determination of the proximate composition of lyophilized green proteins samples, the following AOAC protocols were used (AOAC International 2016): (I) moisture was analyzed using a hot air oven and a dried leaf sample until constant weight (AOAC 952.08, 2016); (II) total fat was examined according to the acid hydrolysis method by utilizing a Soxhlet extractor and drying until a constant weight (AOAC 948.15, 2016); (III) the ash was evaluated according to the gravimetric method, which involved incinerating a leaf sample at 550 °C (AOAC 930.30, 2016); (IV) the dietary fiber was determined according to the enzymatic gravimetric method (AOAC 985.29, 2016). Finally, by using the difference approach, the total amount of carbohydrates was computed.

***Amino acid composition***.

The amino acids composition was determined by high performance anion-exchange chromatographic technique coupled with integrated pulsed amperometric detection (HPAEC-IPAD) at the standardized laboratory of SOJAPROTEIN, as described in the study by (Knežević-Jugović et al. 2022).

#### In vitro bioactive analysis of crude green proteins

The bioactivity of crude green proteins was analyzed in terms of their antioxidant capabilities, namely their electron transfer ability, using the ABTS radical scavenging method, and their ability to chelate ferrous ions as detailed in the study by Knežević-Jugović et al. 2022. Prior to the analyses, the lyophilized green protein powder was dissolved in water to achieve a protein concentration of 1–6 mg/mL and hydrated on a magnetic stirrer (IKA Magnetic Stirrers C-MAG HS 7 with ETS-D6, IKA-Werke GmbH & Co. KG, Germany) for 60 min at 250 rpm, at room temperature. After that, the following analyses of antioxidative activity were conducted.

The ability of green proteins to transfer electrons was analyzed using the ABTS scavenging method. The reagents were prepared according the previously described protocol, where 0.01 mL of the protein solution was mixed with 1 mL of freshly prepared ABTS radical cation solution. After incubation at room temperature for 5 min in the dark, absorbance was measured at 734 nm. The percentage of ABTS inhibitory activity was then expressed as the ratio of the difference in absorbance between the control and the sample to the absorbance of the control. The results were expressed as mmol of Trolox equivalents per gram of protein (mmol TE/g protein).

The ability of green proteins to chelate ferrous ions was also investigated. Briefly, 0.15 mL of the prepared protein solution was mixed with 0.6 mL of water and 0.075 mL of 2 mM iron (II) chloride, and incubated for 3 min. To initiate the reaction, 5 mM ferrozine was added, and after mixing, the solution was incubated for the next 10 min. Then, absorbance was measured at 562 nm, and the degree of chelating activity was calculated as the ratio of the difference in absorbance between the control and the sample to the absorbance of the control. The results were expressed as mmol of EDTA equivalents per gram of protein (mmol EDTA/g protein).

To determine the reaction kinetics for both methods, the effect of reaction time on the percentage of inhibition was examined, in addition to the standard assay times. For ABTS radical scavenging activity, reaction times of 1, 2, 3, 4, 5, 7, 10, 15, and 30 min were analyzed, and for Fe^2+^ ion chelation, reaction times of 1, 2, 3, 5, and 10 min were evaluated. All procedures were carried out as previously described, using protein concentrations ranging from 1 to 6 mg/mL.

### Evaluation of green proteins solubility

The protein solubility was performed as described, with some modifications (Osman 2004). Crude green protein concentrate was dissolved in water to obtain a protein concentration of 7 mg/mL. The resulting dispersion was then stirred with a magnetic bar (IKA Magnetic Stirrers C-MAG HS 7 with ETS-D6, IKA-Werke GmbH & Co. KG, Germany) at 250 rpm for 60 min. After that, the pH of the protein solutions was adjusted with 2 M NaOH and 2 M HCl. The solutions were diluted to a protein concentration of 5 mg/mL, followed by stirring with a magnetic bar at 250 rpm for 30 min. After hydration, the solutions were centrifuged (MiniSpin^®^, Eppendorf, Hamburg, Germany) at 12,000 rpm for 10 min at room temperature. The supernatants were collected, and protein solubility was determined as the percentage of the soluble proteins (supernatant) in relation to the initial protein concentration. Protein content was estimated by the modified Lowry method, while all samples were analyzed in duplicate and measured in triplicate. The solubility of both native and modified proteins was determined using the same method at a standard temperature of 20–25 °C and a constant protein concentration of 5 mg/mL.

#### Effect of salt concentration on solubility of green proteins

The lyophilized protein powder obtained under optimal pH-shift condition was dissolved in NaCl solution of specific concentrations (0.25, 0.5, 0.75 and 1 M). After that, the protein solubility was determined using the same method, as previously described. The only difference was that, instead of dilution with water, NaCl solution of specific concentrations were used, as described by (Tanger et al. 2021).

### Measurement of zeta potential of green proteins

The green protein powder was dissolved in water and stirred with a magnetic stirrer (IKA Magnetic Stirrers C-MAG HS 7 with ETS-D6, IKA-Werke GmbH & Co. KG, Germany) at 250 rpm for 60 min to ensure full hydration. Then the sample was diluted with non-ionized distilled water to reach a protein concentration of 1 mg/mL. For zeta potential measurements, a Zetasizer Nano ZS device associated with the Mastersizer 2000 software package and automatic titrator (version 6.12; Malvern Instruments Ltd., Malvern, UK) was used. All measurements were conducted in triplicate, at a standard temperature of 20–25 °C, and the average zeta potential values were calculated.

### Modification of green proteins by pH-shift treatment

Crude green protein concentrate was dissolved in water to obtain a protein concentration of 10 mg/mL, and hydrated on a magnetic stirrer (IKA Magnetic Stirrers C-MAG HS 7 with ETS-D6, IKA-Werke GmbH & Co. KG, Germany) at 250 rpm for 60 min. The dispersion was then adjusted to alkaline pH (pH 11 and pH 12) with 2 M NaOH and further stirred for 30 min. The resulting solution was incubated in a water bath for 5, 30, 60 and 120 min at a temperature of 20, 60, 70, 80 °C. Afterwards, the solution was immediately cooled until it reached room temperature. The final pH was then adjusted to a neutral and slightly alkaline (pH 7 and pH 8) environment. If necessary, water was added to obtain a final protein concentration of 5 mg/mL (Jiang et al. 2009).

#### Optimization of the pH-shift treatment for green proteins

Green protein powder was subjected to selected pH-shift treatment conditions. The green powder was resuspended in water to obtain a protein concentration of 35 mg/mL, and was hydrated on a magnetic stirrer (IKA Magnetic Stirrers C-MAG HS 7 with ETS-D6, IKA-Werke GmbH & Co. KG, Germany) for 60 min. The solution was then adjusted to pH 12, using 2 M NaOH, and was additionally hydrated for 30 min. Afterwards, the solution was incubated in a water bath at 80 °C and 70 °C for 30 min and at 60 °C for 60 min. The solutions were then immediately cooled, and the pH was adjusted to pH 8 with 2 M HCl. The solutions were diluted to a protein concentration of 25 mg/mL, mixed on a magnetic stirrer for 30 min at 250 rpm, and then lyophilized (same conditions as for the isolation process) and further characterized.

#### SDS-PAGE electrophoresis of green proteins

The electrophoresis was performed as described, with some modifications (Laemmli 1970). Both, the selected protein samples modified by pH-shift treatment and the control (native sample) were subjected to SDS-PAGE. After pH-shift treatment, the protein samples were centrifuged (MiniSpin^®^, Eppendorf, Hamburg, Germany) at 12,000 rpm for 10 min to obtain the soluble protein fraction (supernatant). The obtained samples were then diluted to achieve the same final protein concentration as the native sample. SDS-PAGE buffer, with and without a reducing agent, was added to all protein samples in a ratio of 1:1, and mixed. For the reducing conditions, samples were boiled for 5 min and then immediately cooled. The prepared samples were loaded into a 12% acrylamide gel (mPAGE™ 12% Bis-Tris Precast Gel; 10 × 8 cm, 12-wells; Merck KGaA, Darmstadt, Germany), and after staining with Coomassie Brilliant Blue R250 dye and decolorizing, they were compared against a protein marker.

#### Average particle size determination

After the selected pH-shift treatment, total and soluble protein samples were separated by centrifugation at 12,000 rpm for 10 min at room temperature. Both sample types were then diluted with non-ionized distilled water to a final protein concentration of 1 mg/mL. The diluted samples were stirred on a magnetic stirrer (IKA Magnetic Stirrers C-MAG HS 7 with ETS-D6, IKA-Werke GmbH & Co. KG, Germany) at 250 rpm for 60 min before analysis. Then, 1 mL of the sample was placed into a cuvette and the average particle size was measured using a Zetasizer Nano ZS device associated with the Mastersizer 2000 software package (version 6.12; Malvern Instruments Ltd., Malvern, UK). All measurements were done in triplicate and the results were reported as the averages of these replicates.

#### Fourier-transform infrared spectroscopy

FTIR analysis of both native and pH-shift modified green protein powders was performed in ATR mode using an IRAffinity^− 1^ spectrometer (SHIMADZU, Kyoto, Japan). The samples were scanned 100 times with a resolution of 4 cm^− 1^, in the IR range of 500–4000 cm^− 1^. The spectra of green proteins samples were performed in triplicate and the results were reported as the averages of these replicates (relative standard deviation < 5%). The amide I region (1600–1700 cm^− 1^) was used to analyze the secondary structure of green membrane proteins. The Fit Peaks Pro function (OriginPro 9.0 software) was used to perform deconvolution of amide I bands, including baseline subtraction, second derivative, smoothing with the Savitzky-Golay function, and peak fitting using the Gaussian function.

To purify green protein powder for more accurate secondary protein structure determination, the following isolation steps were performed: After thermal coagulation, the resulting green pellet was resuspended in distilled water and centrifuged at 7830 rpm for 10 min at room temperature. This washing step was repeated three times to remove impurities. The final green pellet was then collected and lyophilized. The resulting powder was subjected to a pH-shift treatment under optimized conditions (70 °C for 30 min) as previously described (at Materials and Methods, section Optimization of the pH-shift treatment for green proteins). After pH adjustment and mixing, the resulting soluble protein fraction was separated by centrifugation at 6000 rpm for 15 min at room temperature to further eliminate insoluble impurities and protein aggregates. The collected supernatant was lyophilized and analyzed by FTIR under the same conditions as previously described.

#### Protein staining, microscopic images

The initial protein solution at a concentration of 1 mg/mL was prepared from the selected samples. Then, aliquots of 100 µL of the diluted samples were combined with Coomassie Brilliant Blue R250 solution. Following the staining and aggregation, the samples were fixed and observed under a microscope (Motic BA210, Xia-men, China) with a Monticam digital camera (1SP, 1.3 MP) and the magnification of 400. The images were processed using Motic Images Plus 2.0 software.

### Statistical analysis

Triplicate measurements were taken and the final results were expressed as mean ± standard deviation (SD). The statistical analysis of the data was performed using Libre office software (OpenOffice.org), while for the FTIR deconvolution OriginPro9.0 software (Origin Lab Corporation, Northampton, MA, SAD) was used. A comparison of the means was ascertained by Tukey’s test at 5% significance level using one-way analysis of variance (ANOVA).

## Results and discussion

### Isolation of pumpkin leaf proteins and compositional analysis of crude green proteins

**Fig. 1:**
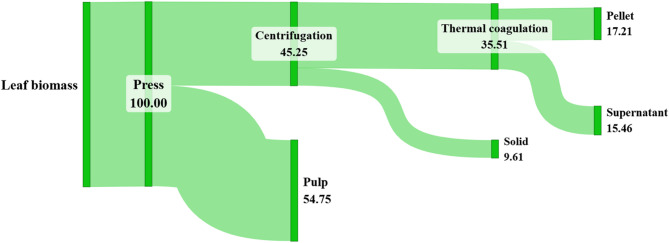
Flow diagram of protein distribution (%) throughout the isolation steps. The output fractions of the isolation are represented by curved lines, while the straight lines represent the desired fractions

To gain a comprehensive understanding of the isolation process, it is essential to examine all input and output fractions, along with the final overall outcome of the isolation process. Therefore, in this research, it is crucial to assess the protein content at each isolation step in order to determine the protein distribution throughout the process. The protein mass balance during the isolation of pumpkin leaf proteins by thermal coagulation is illustrated in the Sankey diagram (Fig. 1).

The modified Lowry method was selected for protein determination due to its practicality, as a relatively fast spectrophotometric protein assay (except for the pulp, obtained by subtracting the content of the leaves and the juice). The results demonstrate that the modified Lowry method (29.24% for leaf and 56.23% for green powder protein content) is reliable, as the findings did not significantly deviate from the expected values estimated by the Dumas method, as shown in Table 1. It is important to note that the more commonly used protein assay, the Bradford method, was also examined, but it was found that the protein concentration deviated significantly from expected values. This can be explained by the interference of Coomassie blue dye with lipids, and therefore, it is an unsuitable method for membrane protein determination (Kirazov et al. 1993).

**Table 1:** Chemical composition of pumpkin leaf and crude green protein powder, % dry matter

Chemical composition	Dry pumpkin leaf (wt%)	Dry crude green protein powder (wt%)
Protein (Nx6.25)	28.63 ± 1.40	53.58 ± 2.68
Lipids	3.62 ± 0.18	5.93 ± 0.30
Cellulose	6.31 ± 0.32	3.19 ± 0.16
Ash	9.22 ± 0.46	7.1 ± 0.36
Fiber	18.09 ± 0.9	7.18 ± 0.36
Carbohydrate	28.44 ± 1.42	18.39 ± 0.92
Dry matter	93.90 ± 3.69	94.76 ± 3.74

The first step in leaf protein biorefinery is the separation of the leaf juice (protein-enriched fraction) from the lignocellulosic fraction (i.e., pulp), achieved by mechanical pressing using a twin-screw press. It is extremely important to emphasize that the first step of isolation contributed to the greatest protein yield losses, with approximately half of the total protein (54.75%) remaining in the pulp. Consequently, this isolation step requires further optimization, as significant protein loss was observed. One potential solution to increase the final protein yield, as suggested by previous studies, is to re-press the pulp (Tenorio et al. 2016; Santamaría-Fernández and Lübeck 2020).

After pressing, the remaining solid fraction in the juice was removed by centrifugation. A low centrifuge speed has been applied to preserve the proteins in the juice. This step resulted in slight protein loss, with only 9.61% of the total protein precipitating. It could be concluded that these proteins are either unstable, as demonstrated by their tendency to aggregate, or they are part of the plant cell that was not mechanically disrupted.

The purified juice was then heated to separate the white and green protein (byproduct) fractions, which is a common method for isolating white proteins (Nieuwland et al. 2021). After thermal coagulation followed by centrifugation, the protein distribution in the supernatant and precipitate was almost equal (43.54% and 48.48% respectively). This finding aligns with previous research, which also confirmed the same protein ratio (Tenorio et al. 2016). Since approximately half of the proteins are considered waste, further analysis of this byproduct is essential to ensure the complete utilization of leaf biomass.

The obtained green pellet was not purified from the residual white protein fraction in order to enhance the final yield of white protein fraction, as significant protein loss was observed during repeated rinsing with water (results not shown). Furthermore, this step is typically not performed to increase the yield of white proteins, likely due to the residual presence of green pigment. Therefore, a certain percentage of white proteins is expected to remain in pellet, which is beneficial for the final crude green protein powder, as it becomes further enriched with proteins, while reducing water consumption during the isolation process.

It is important to emphasize that, in this study, crude/unpurified green proteins were analyzed to gain a comprehensive understanding of this protein fraction. The results showed that 17.21% of the total leaf proteins remained in the green protein pellet, representing 48.48% of the overall proteins in the leaf juice. The isolated crude green protein powder contained 53.58% protein, based on a dry weight basis, with the detailed chemical composition presented in Table 1. Based on the Dumas method, it was estimated that 47.95 g of crude green proteins could be produced from 1 kg of dry leaves. In addition to proteins, carbohydrates are the second most abundant component in the green protein powder, accounting for 18.39% of the dry matter. Thus, in order to purify the green protein powder, the removal of carbohydrates, specifically fibers, is essential.

#### Fourier-transform infrared spectroscopy of crude green protein powder

**Fig. 2:**
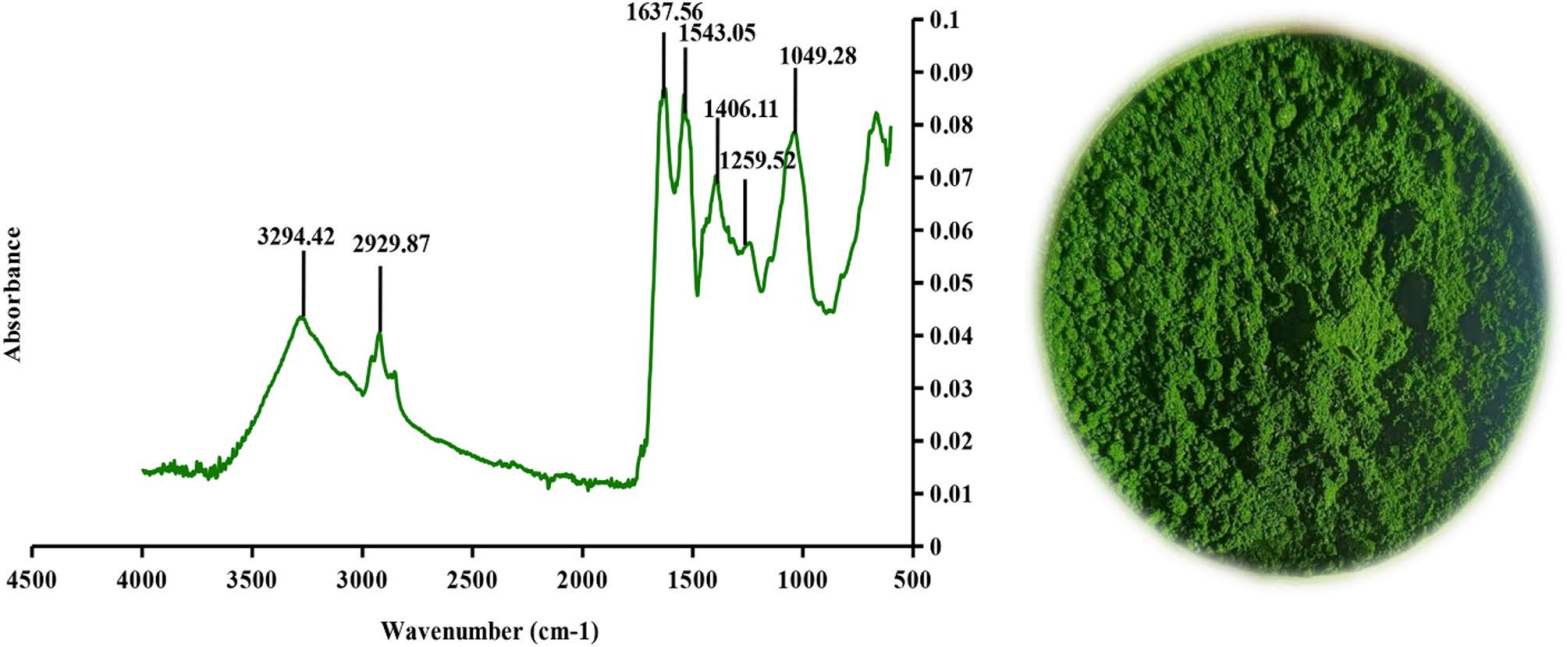
FTIR spectra and image of the crude green protein powder. The prominent peaks are highlighted on the spectrum

Further compositional analysis of the obtained green protein powder was analyzed by infrared ATR-FTIR spectroscopy (4000–500 cm⁻¹), and the corresponding spectrum is shown in Fig. 2. It can be observed from the highlighted peaks that the crude green protein powder exhibited characteristic bands typically associated with protein concentrates.

The protein bands were located at 1637.56 cm^− 1^ (Amide I) and 1543.05 cm^− 1^ (Amide II), corresponding to the C = O stretching vibration and the N-H bending vibration, respectively (Parker 1971; Barth 2007). Furthermore, the third characteristic protein band (amide A), resulting from N-H stretching vibration, was detected at 3294.42 cm^− 1^, and the amide III band can also be observed around 1406.11 cm^− 1^ as a small intensity peak (Barth 2007).

Apart from the protein bands, major carbohydrate peaks were detected in the region of 1250 –800 cm^− 1^, which was consistent with the composition of the crude green protein powder (see Table 1). Plant biomass exhibited characteristic carbohydrate bands associated with the presence of fibers (Rafidison et al. 2020; Zhongqi et al. 2022). A pronounced peak at 1049.28 cm^− 1^, along with a band of lower intensity at 1259.52 cm^− 1^, can be attributed to C-O vibrations of cellulose and hemicellulose, respectively (Akhtar et al. 2016; Akwu and Singh 2019). Moreover, previous studies have shown that an absorption band in the region of approximately 1605 cm⁻¹ (Kostryukov et al. 2023) to 1635 cm⁻¹ (Akhtar et al. 2016) can be attributed to the asymmetric stretching vibrations of the carboxylate anion in hemicellulose, as well as deformation vibrations of H–O–H in absorbed water. This spectral overlap is important to consider, as it may affect the accurate interpretation of the amide I protein band, which is critical for determining secondary protein structure (as explained in detail in the Results and Discussion, section FTIR- spectroscopy and amide I bend deconvolution in the structural analysis of green proteins).

Finally, the absorption band at 2929.87 cm^− 1^ corresponded to -CH_2_ asymmetric stretching vibrations of the methylene group of lipids (Akwu and Singh 2019).

#### Amino acid profile of the green protein powder

The amino acid profile is crucial for evaluating the nutritional value of the novel proteins. In order to compare the amino acid profile of green proteins with other plant-based proteins, as well as the standard recommended values by FAO/WHO (Vera-Salgado et al. 2022), the obtained amino acid content was expressed in relation to the protein mass of the crude green protein powder (mg/g protein), as shown in Table 2.

The prominent amino acids in terms of quantity were glutamate, aspartate, and leucine, followed by arginine and lysine. The crude green protein powder was enriched with a complete profile of essential amino acids, as recommended by the FAO/WHO (Vera-Salgado et al. 2022). It is important to emphasize that the intake of threonine and phenylalanine were approximately twice the standard value, making them the main essential amino acids in the crude green protein powder. Furthermore, most amino acids were present at higher levels, except for the sulfur-containing amino acids and histidine, which were close to the reference values.

It is important to note that the amino acid composition of crude green proteins is comparable to soy protein, a representative example of plant-based proteins, giving them a significant advantage as a new protein source (Hughes et al. 2011). Additionally, it is essential to consider that the obtained crude green protein powder likely contains some percentage of soluble proteins, as the green pellet was not washed during the isolation process. Moreover, the high essential amino acid content was, to some extent, perhaps part of the Rubisco and other soluble proteins, as studies have shown that Rubisco offers exceptional nutritional value as a protein (Nieuwland et al. 2021). Furthermore, the well-balanced amino acid composition of green proteins can be further enhanced by purifying the obtained crude green protein powder. This is suggested by the protein content, which was only 53.58%, with the amino acid values expressed in relation to the mass of the crude green protein powder (mg/g powder) in Table 2.

Overall, the crude green protein powder exhibited surprisingly high nutritional value, especially considering it is a waste product from leaf biomass, making it a promising potential protein source.

**Table 2:** Amino acid composition of the crude green protein powder

Total amino acid	mg/g protein	FAO/WHO Adults, mg/g protein	mg/g powder
Lys Ala Thr	**71.1** 58.9 **41.2**	**45** **23**	4.02 ± 0.31 3.33 ± 0.67 2.33 ± 0.47
Gly	44.2		2.50 ± 0.50
Val	**58.5**	**39**	3.31 ± 0.66
Ser	44.5		2.52 ± 0.50
Pro lle Leu His Glu Asp Arg	43.5 **51.1** **83.3** **19.1** 123.6 93.2 72.5	**30** **59** **15**	2.46 ± 0.49 2.89 ± 0.58 4.71 ± 0.94 1.08 ± 0.22 6.99 ± 1.40 5.27 ± 1.05 4.10 ± 0.80
Met + Cys	**27.9**	**22**	1.59 ± 0.31
Phe + Tyr	**88.8**	**38**	5.02 ± 0.90

*mg/g protein represents the milligrams of amino acid per gram of protein in crude green protein powder

**mg/g powder represents the milligrams of amino acid per gram of crude green protein powder

#### In vitro assessment of the antioxidant activity of crude green protein powder

Antioxidant activity is a very important parameter when testing the quality of a new protein. It has been shown that proteins can inhibit lipid oxidation; thereby, as additives, they can significantly extend the shelf life of the product, thus increasing its stability (Elias et al. 2008). It is well known that proteins from leaves possess biological activity, as evidenced by the antioxidant properties of numerous leaf extracts (Youn et al. 2019; Vera-Salgado et al. 2022). However, it should be noted that this is preliminary research involving unpurified green proteins, and as such, the obtained results provide the initial assessment of their antioxidant capacity. Furthermore, given that a certain contribution of phenolic compounds, as well as pigments, is expected, the obtained antioxidant activity should be considered as the overall effect of the isolated powder.

Therefore, the further approach of this research is the analysis of the antioxidant capacity of the obtained crude green protein powder, through two analyses selected for their different principles of action: radical neutralization (ABTS) and ferrous ion chelating ability, as shown in the Table 3. Given that the tested methods for evaluating the antioxidative activity of crude green proteins are demonstrated to be time-dependent processes, Table 3 presents the inhibition values ​​recorded at the point when the reaction plateau was reached, while the corresponding graphs in Supplementary material (Figure S4) illustrate the kinetic behavior of the reaction.

The ABTS radical scavenging activity of crude green proteins demonstrated increased inhibition with higher protein concentrations, reaching 98.39% at a protein concentration of 6 mg/mL, which also resulted in shorter reaction times. According to the Figure S4, the standard 5-minute incubation time commonly applied in the ABTS radical scavenging assay was sufficient for samples with elevated protein concentrations (5 and 6 mg/mL). However, starting from approximately 4 mg/mL, substantial increases in inhibition were observed, with the reaction time extending to 10 min, which was more pronounced at lower protein concentrations. Accordingly, a 10-minute reaction time was defined as optimal for these concentrations, as the reaction reached a plateau by this point. The extended reaction time could be associated with the lower protein concentration analyzed, as well as a potentially increased relative effect of impurities, given that proteins constitute nearly half of the crude green protein powder. Furthermore, when compared with Trolox as a standard, the antioxidant capacity of the crude green proteins was 107.64 mmol TE/g protein at 5 min of reaction, and 143.95 mmol TE/g protein at the plateau (10 min), measured at the lowest analyzed protein concentration of 1 mg/mL.

The ability of crude green proteins to chelate ferrous ion proved to be equally effective. The protein concentration limit for metal chelation was 2 mg/mL, and the maximum inhibition, for the tested protein concentration, was at 6 mg/mL, reaching 79.66%. In contrast to the ABTS method, prolonged reaction time in this assay did not significantly affect the results at the tested protein concentrations. As shown in the figure, the reaction kinetics increase up to the literature-defined reaction time of 3 min, after which the Fe^2+^ ion chelating ability remains constant. This indicates that Fe^2+^ ion chelation by the green proteins is not time-dependent. Furthermore, as demonstrated in Table 3, lower protein concentrations were insufficient to achieve significant Fe^2+^ ion chelation. Therefore, higher protein concentrations are required to observe a substantial chelating effect. The ability of crude green proteins to chelate Fe^2+^ ions was expressed using EDTA as the standard. For a protein concentration of 2 mg/mL, the obtained results were 0.856 mmol EDTA/g protein.

Additionally, EC_50_ values were calculated for both antioxidant assays as important parameters, serving as quantitative indicators of antioxidant capacity. Using logarithmic regression analysis of inhibition values versus protein concentration at 10 and 3 min for the ABTS and Fe^2+^ assays, respectively. The EC_50_ for ABTS was determined to be 1.244 mg/mL, with the corresponding regression model: y = 37.589 ln (x) + 41.883 (R² = 0.999), and for Fe²⁺ ion chelation, the EC_50_ was calculated to be 2.779 mg/mL, with the regression model: y = 39.056 ln (x) + 10.084 (R² = 0.953). These results indicate that, under the tested conditions, green proteins exhibit stronger radical scavenging activity than Fe^2+^ ion chelating ability, as the EC_50_ values ​​for the ABTS assay were lower compared to those for the Fe^2+^ ion chelation assay.

Considering that the obtained crude green protein powder is unpurified, it exhibited favorable antioxidant activity. Furthermore, the antioxidant capacity of green proteins might be improved through protein hydrolysis, as peptides generally exhibit significantly higher antioxidant activity than intact proteins (Elias et al. 2008).

**Table 3:** The antioxidative capacity of the crude green protein powder

Protein concentration, (mg/mL)	ABTS^+^ scavenge ability, (%)	Fe^2+^ ion chelating ability, (%)
1	42.02 ± 2.16	n.d.
2	67.33 ± 3.50	33.70 ± 1.52
3	83.99 ± 1.72	57.94 ± 2.13
4	93.65 ± 3.42	66.76 ± 2.34
5	94.69 ± 2.61	69.32 ± 2.89
6	98.39 ± 2.96	79.66 ± 1.97

* Reaction time in the ABTS radical scavenging assay: 10 min for 1–4 mg/mL and 5 min for 5–6 mg/mL protein concentrations

** Reaction time in the Fe²⁺ ion chelating ability assay: 3 min for all tested protein concentrations

### Solubility and ζ-potential of the isolated green proteins

Protein solubility is a fundamental analysis when testing new proteins, as higher protein solubility is essential for broader applications. However, understanding protein solubility is complex, as it depends on various factors such as amino acid composition, molecular weight, and external environmental conditions like the presence of salts, which will be examined later in this study. The principle behind protein solubility analysis is to evaluate the solubility trend when exposed to different pH values of the medium, typically within the range of pH 2 to pH 12 (Zayas 1997).

**Fig. 3:**
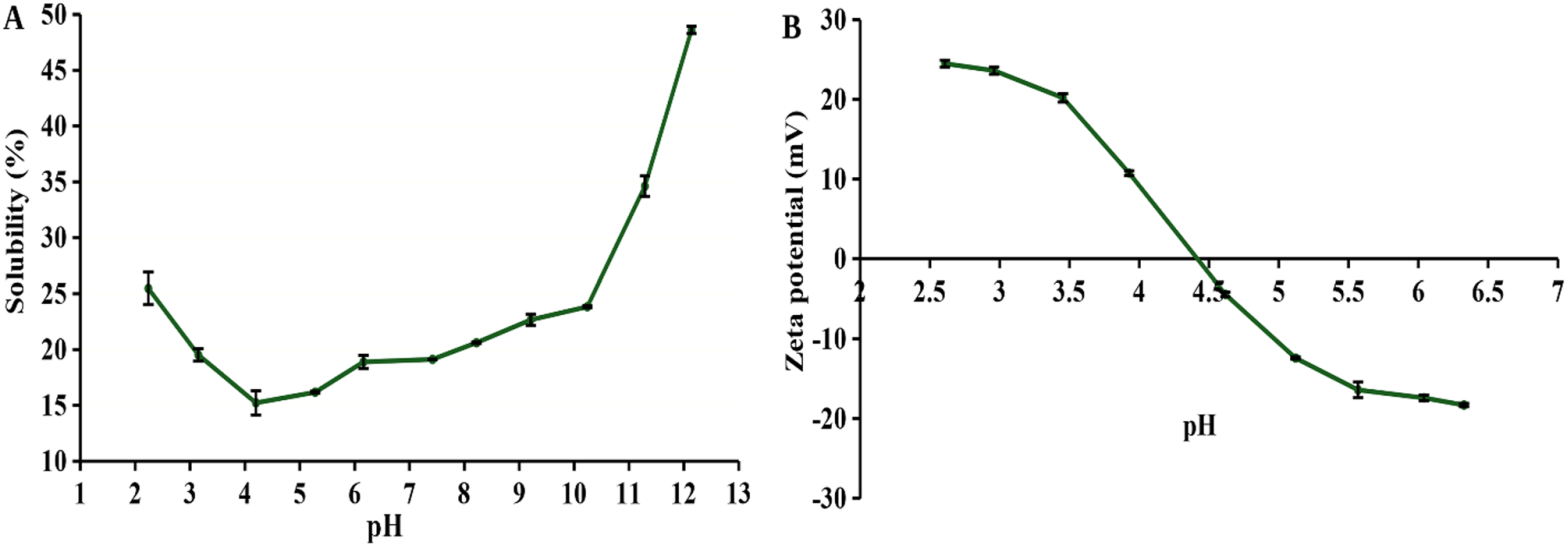
Effect of pH on **(A)** protein solubility and **(B)** ζ-potential of the green proteins. Reaction conditions: concentration = 5 mg/mL, temperature 20–25 °C, native green protein without pH-shift treatment

The protein solubility and ζ-potential of the produced crude green protein powder are shown in Fig. 3. The obtained crude green proteins exhibited a typical U-shaped curve, which is characteristic of most plant proteins (Grossmann and McClements 2023; Alavi et al. 2021). The pH-solubility profile revealed quite low protein solubility, with a maximum of only 48.62% in a highly alkaline environment. Interestingly, the green protein exhibits low solubility across a broad pH-range, from two to ten (below 25%), compared to other plant proteins, that typically cover a narrow range, from four to six (Grossmann and McClements 2023). As a result, the application of green proteins is constrained, as high protein solubility is critical for many applications, especially for incorporation into food products (Zayas 1997). Furthermore, the lowest solubility point was recorded at pH 4 (15.22%), which corresponded with the ζ-potential. According to Fig. 3(B), the isoelectric point was determined to be at pH 4.4, indicating a zero net charge of the proteins. Interestingly, the isoelectric point did not significantly deviate from the previously obtained result by (Tenorio et al. 2017), despite the differences in the protein isolation method.

Since the low solubility of green proteins significantly limits their applications, the next focus of this research will be on enhancing their solubility. This will be accomplished through a customized pH-shift method, specifically adapted to improve the functionality of green proteins.

### pH-shift treatment for enhancing green protein solubility

#### Protein solubility improvement through pH-shift treatment

**Fig. 4:**
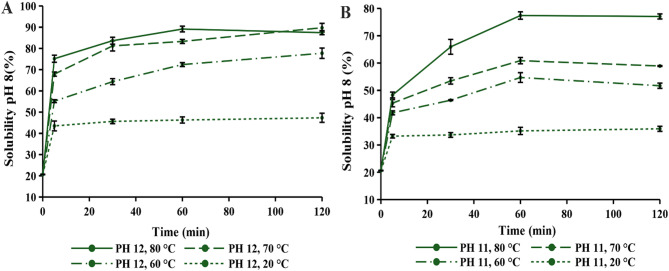
Effect of temperature on protein solubility at pH 8 under alkaline pH of **(A)** 12 and **(B)** 11

**Fig. 5:**
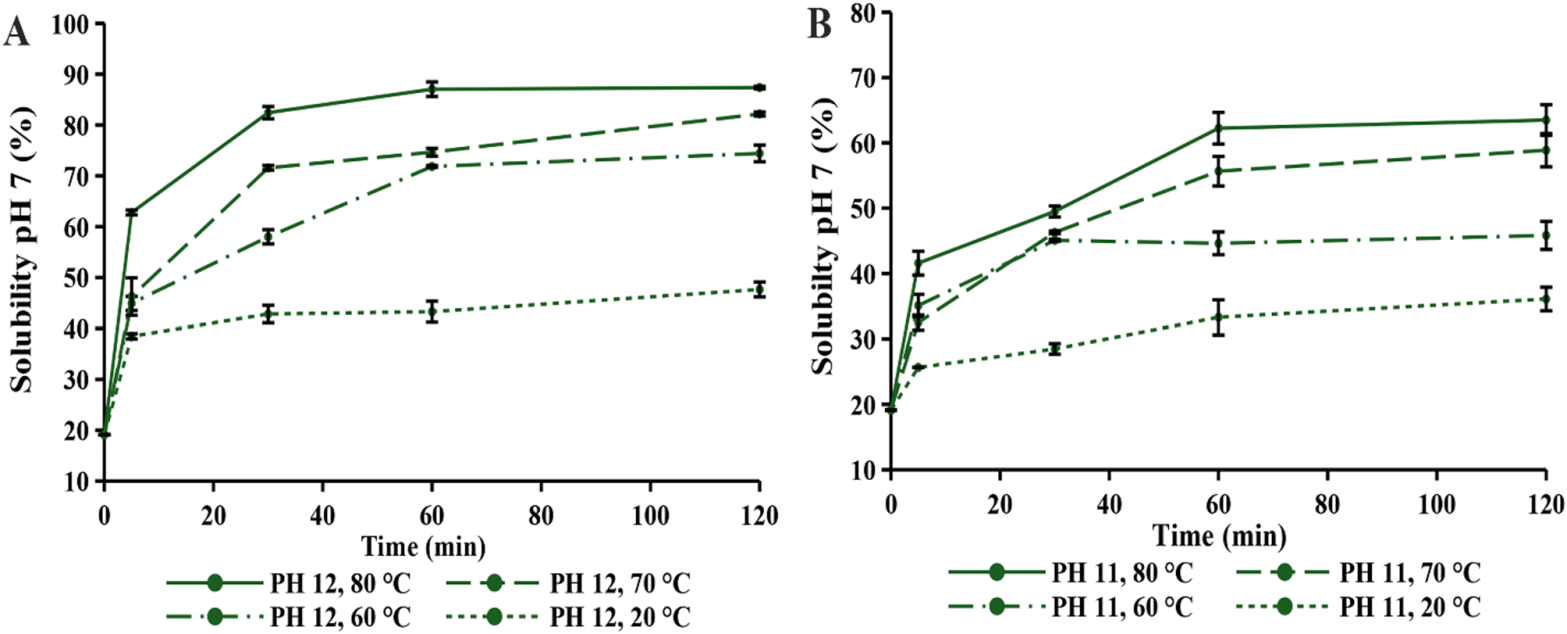
Effect of temperature on protein solubility at pH 7 under alkaline pH of **(A)** 12 and **(B)** 11

The impact of an alkaline environment at pH 12 and pH 11, with varying incubation times and temperatures, on protein solubility is shown in Figs. 4 and 5. The solubility values measured at time zero served as the control, representing the native protein sample before any pH-shift treatment was applied. To assess the effect of the pH-shift treatment, protein solubility was examined at pH 7 and pH 8, as these are commonly used final pH values in similar treatments (Tang et al. 2021).

The impact of a highly alkaline environment on protein solubility was analyzed at room temperature (20 °C) to assess the specific effect of high pH without temperature elevation. The results revealed equivalent maximum solubility at pH 7 and 8, with a shorter incubation time required for pH 8. The solubility has increased relative to the control, but the values still remained quite low. Furthermore, no significant changes in protein solubility were observed even with prolonged incubation for 24 h (results not shown). Following this, the effect of temperature on protein solubility was subsequently tested.

The highly alkaline environment, combined with heat, resulted in a remarkable improvement in protein solubility, reaching 89.74% (Fig. 4(A). Interestingly, Wang et al. (2018) achieved an even more drastic increase in protein solubility through modification of hemp seed protein, despite its initial insolubility being comparable to that of green protein. This also confirms that the same treatment conditions can produce varying effects on protein solubility, as the outcome primarily depends on the inherent structure of protein. In this study, lower pH values were also tested, but even at pH 10, a significantly smaller increase in protein solubility was achieved (results not shown). The same trend was observed by Nissen et al. (2021), who tested an even broader pH range for the modification of alfalfa leaf protein concentrate. Therefore, protein solubility increased with increasing alkalinity of the environment. This was evident even at pH 11, where high protein solubility (77.07%) could only be achieved at higher temperature and with prolonged incubation time (Fig. 4(B), 80 °C for 60 min, at pH 8). Conversely, under other treatment conditions at pH 11, protein solubility remained below 65%, which was more pronounced at pH 7 (Fig. 5).

With an increase in temperature (60–80 °C), protein solubility also increased. However, no specific trend was observed among four treatment conditions. Interestingly, nearly identical protein solubility was obtained at 70 °C and 80 °C for pH 8 (Fig. 4(A), indicating that it reached a plateau. A similar conclusion can be observed from the results presented by Wang et al. (2018), revealing a similar trend at the final pH of 7. On the other hand, for green proteins, pH 7 showed significantly lower protein solubility at 70 °C (Fig. 5(A). Furthermore, at 60 °C, quite similar protein solubility was observed for both final pH values (pH 7 and 8).

The incubation time required to reach a constant solubility value at pH 7 and pH 8 was affected by the alkaline environment (pH 11 and pH 12). At pH 12, a constant solubility was achieved after 30 min at temperatures 70 and 80 °C, whereas at pH 11, 60 min of incubation were required. It can be concluded that protein extraction at pH 11 required a longer duration, which corresponded with the overall lower protein solubility observed.

The above results suggest that alkaline extraction should be performed at pH 12, as the aim of this research is to improve green protein solubility. Furthermore, treatment conditions under which protein solubility (at pH 7 and 8) became almost constant with incubation time were further analyzed for all tested temperatures (80 °C for 30 min, 70 °C for 30 min and 60 °C for 60 min). The reason behind the selected treatment conditions was that further incubation did not show a significant increase in protein solubility. Additionally, the effect of prolonged incubation time on green proteins is not relevant to this research.

#### Average particle size of green proteins modified by pH-shift treatment

**Fig. 6:**
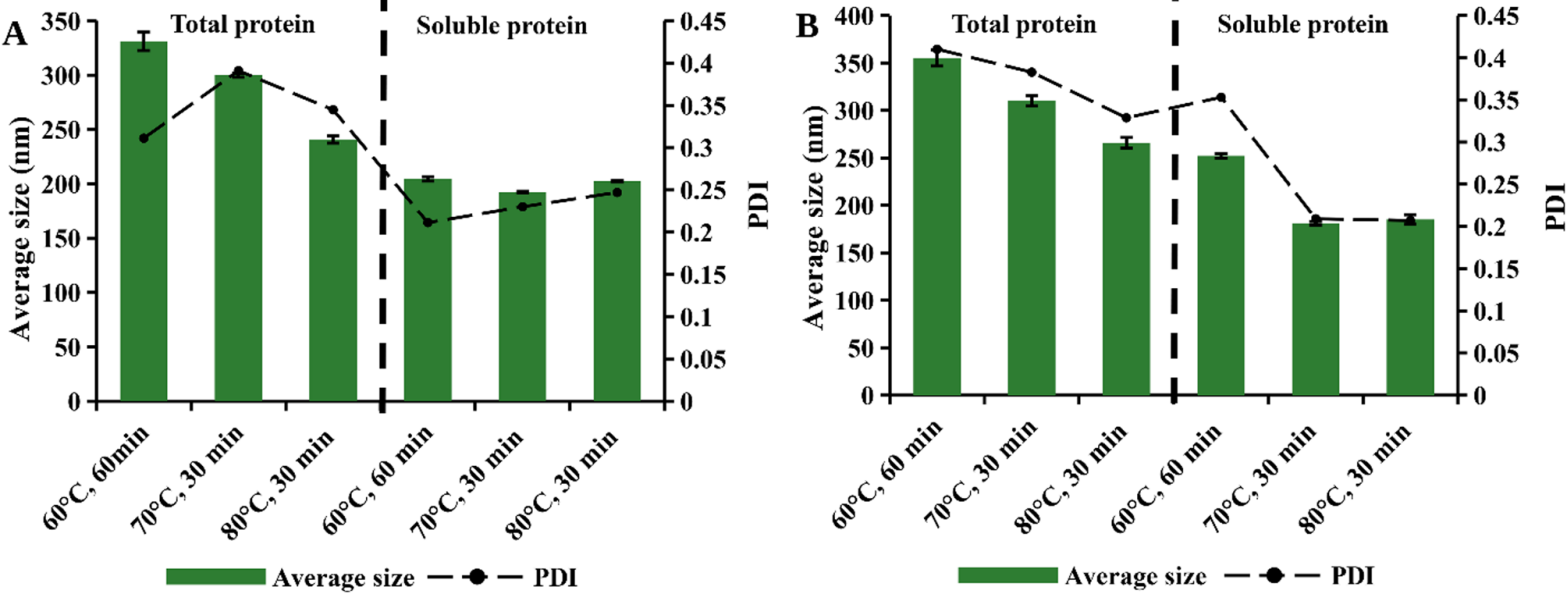
Average particle size and PDI of samples under different pH-shift treatment. Analyzed at final pH **(A)** 8 **(B)** 7. “Total” represents protein samples collected before centrifugation. “Soluble” indicates supernatant fractions collected after centrifugation, representing the soluble protein fraction

Given that solubility was significantly increased compared to the native sample, an interesting subsequent analysis would be to investigate its correlation with particle size distribution. Therefore, the average particle size of green proteins subjected to different pH-shift treatment conditions is shown in Fig. 6. Furthermore, to enable a more detailed evaluation, an additional centrifugation step was incorporated to individually characterize the average particle sizes of the total and soluble green protein fractions under each treatment condition.

The particle size of the native sample (protein without treatment) was significantly larger than that of the treated samples, with an average particle size of 1883 nm and a broad molecular weight distribution (PDI over 0.5). As shown, apart from size reduction, the pH-shift treatment contributed to a more homogeneous system, as the PDI values decreased. This was consistent with Sun et al. (2025), who explained that extremely alkaline conditions induce strong electrostatic repulsion, leading to structural unfolding of proteins, reduced particle size, and a more uniform particle distribution.

The final pH values (pH 7 and 8) had no significant influence on the particle size. However, the particle size of total protein samples was slightly larger at pH 7 than at pH 8. It was observed that temperature had a favorable effect on particle size, as an increase in temperature led to decrease in the average particle size of the total protein samples. On the other hand, the average particle size of soluble proteins (supernatant) remained constant across all treatment conditions, except at 60 °C for pH 7 (Fig. 6(B). After centrifugation, particle size was further reduced to approximately 200 nm, resulting in a more uniform particle size distribution (lower PDI values). This is in agreement with the higher protein solubility demonstrated previously. Other studies have similarly shown a correlation between protein solubility and smaller particle size (Dai et al. 2022; Li et al. 2020). This phenomenon is explained by the fact that as the particle size decreases, the contact surface area with water increases, leading to enhanced protein solubility (Li et al. 2020). Furthermore, a significant reduction in particle size is important, as it is associated with enhanced gelation properties of proteins (Li et al. 2020; Sun et al. 2025).

#### Electrophoresis protein profile of the selected modified green proteins

**Fig. 7:**
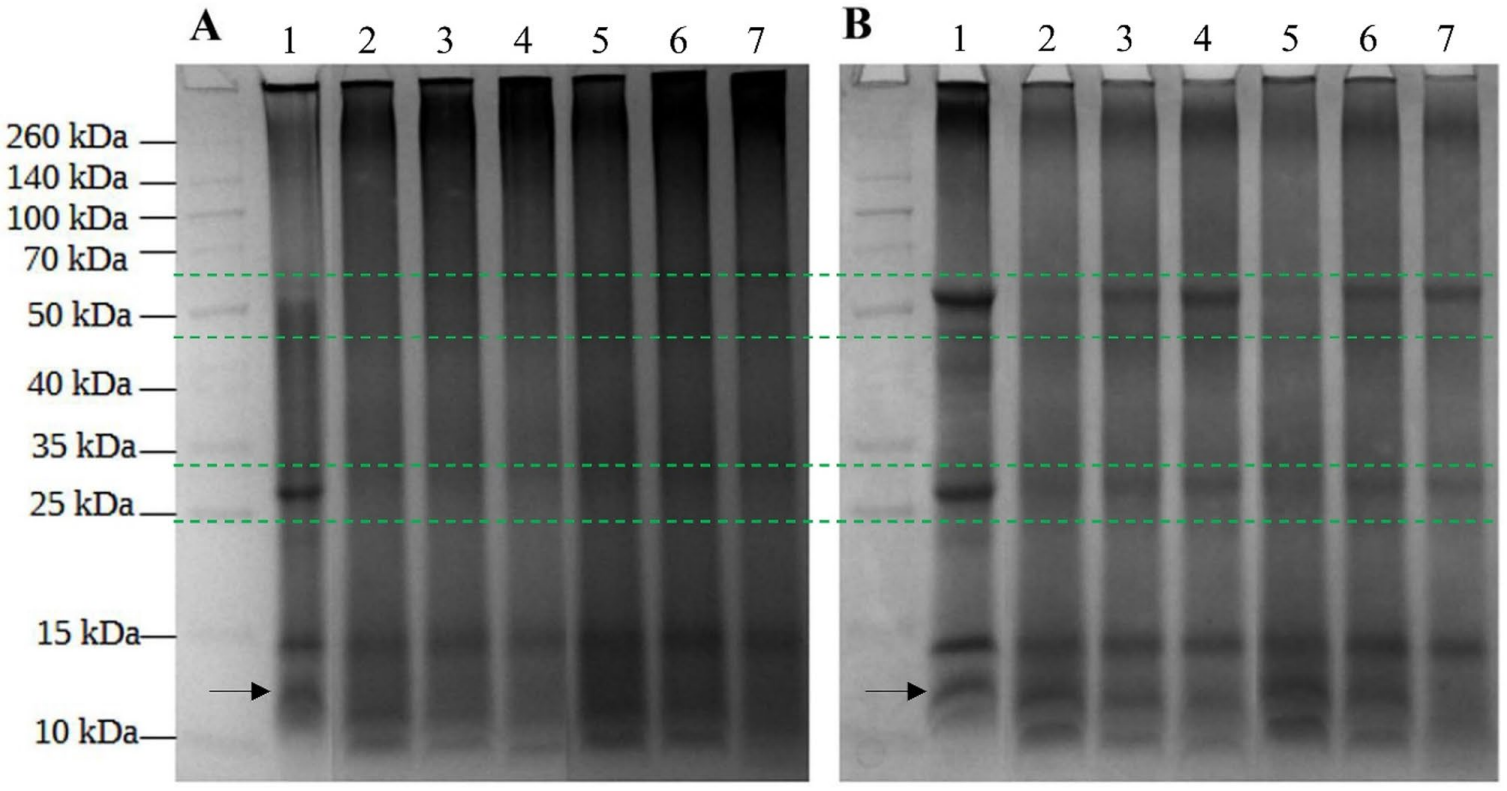
SDS-PAGE patterns of different pH-shift treatment conditions. **(A)** non-reducing **(B)** reducing conditions. Left to right lane (same for both gels): Protein marker; control; supernatant of modified proteins at pH 8: 80 °C for 30 min; 70 °C for 30 min; 60 °C for 60 min; supernatant of modified proteins at pH 7: 80 °C for 30 min; 70 °C for 30 min; 60 °C for 60 min

In order to correlate improved solubility with potential protein structural changes induced by pH-shift modification, electrophoresis was performed. The electrophoresis pattern of soluble proteins (supernatant) under non-reducing and reducing conditions for different pH-shift treatment conditions, compared to the native sample (without centrifugation), is shown in Fig. 7. The supernatant was analyzed to create a profile of soluble proteins and to evaluate the protein extraction potential under various pH-shift treatment conditions. It is important to note that the total protein profile of samples modified by pH-shift treatment (without centrifugation) showed identical band patterns, suggesting that the faint bands observed at approximately 25 kDa and 50 kDa (Marked with a green dashed line, Fig. 7) resulted from protein modification, as demonstrated by the representative treatment conditions in the Supplementary material, Figure S1.

As expected, since the green protein was not rinsed with water during isolation, the residual fraction of white proteins appeared under reducing conditions. Characteristic bands at 50 and 15 kDa can be assigned to the larger and smaller subunits of Rubisco, which are connected by disulfide bonds (Grácio et al. 2023). It can be concluded that thermal coagulation, to some extent, disrupted the structure of Rubisco, as the smaller subunit appeared even under non-reducing conditions, although this could also indicate the presence of another protein.

The distinct band around 25 kDa probably indicates the presence of various green protein subunits that are not well separated, which may result from low protein purity and/or the isolation method, as well as the electrophoresis itself. Subunits such as the antenna complex PS II (CP24, CP26 and CP29) and the light harvesting protein complex PSI and PSll are some examples (Zolla et al. 1999; Suorsa et al. 2014). Two less intense bands between 40 and 50 kDa, can be assigned to chlorophyll binding proteins CP43 and CP47 of Photosystem II (Johnson and Pakrasi 2022). Meanwhile, lower molecular weight proteins around 12 and 10 kDa may be apo-proteins of chlorophyll-proteins or polypeptides that are an integral part of the complex (Bassi and Simpson 1988).

Regardless of treatment conditions, all treated samples appeared equivalent up to 15 kDa with diverse band intensities. The results revealed that the treatment did not alter the overall structure of the analyzed crude green proteins extract, while the primary changes observed were related to the formation of new protein bonds. Compared to control, the intensity of the band at 25 kDa was significantly reduced under non-reducing conditions (Fig. 7A). The observed differences between the control and treated samples suggest that disulfide bonds were likely formed during the pH-shift treatment, as disulfide bonds remained intact under non-reducing conditions. Other plant proteins exhibited the same type of bond formation during pH-shift treatment (Alavi et al. 2021; Wang et al. 2018). Furthermore, other protein interactions may be present, since control exhibited a more pronounced band even under reducing conditions (Fig. 7B). A similar trend was observed at 50 kDa, where a decrease in intensity under reducing conditions was noted.

Treatment condition (80 °C, 30 min, lanes 2 and 5) was too aggressive as the bands at 50 and 25 kDa became barely visible, exhibiting a smearing effect. Interestingly, the bands at 70 °C (lanes 3 and 6) remained stable, while protein solubility remained the same. Compared to control, the band at approximately 12 kDa (Marked with a black arrow, Fig. 7) was quite faint for treatment conditions (60 °C, 60 min, lane 4) at pH 8, and was completely absent at pH 7 (lane 7). This treatment condition was apparently too mild, which is consistent with the lower protein solubility observed. Nevertheless, a slightly alkaline environment could potentially enhance protein extraction.

The quantity of lower molecular weight proteins increased as the treatment conditions became more drastic, leading to the appearance of a third band at 10 kDa. This band could be a result of the pH-shift treatment, since it was not present in the control sample. On the other hand, there may be an issue with pigment accumulation at the bottom of the gel, preventing proper protein separation during electrophoresis, particularly in the control sample.

Previous studies confirmed the formation of aggregates during pH-shift treatment, as evidenced by a dark line upon entering the gel (Wang et al. 2018). It is difficult to say whether the same principle applies to green proteins, as a dark line appeared on both gels for control and treated samples.

#### Further solubility analysis of selected pH-shift treatment conditions

**Fig. 8:**
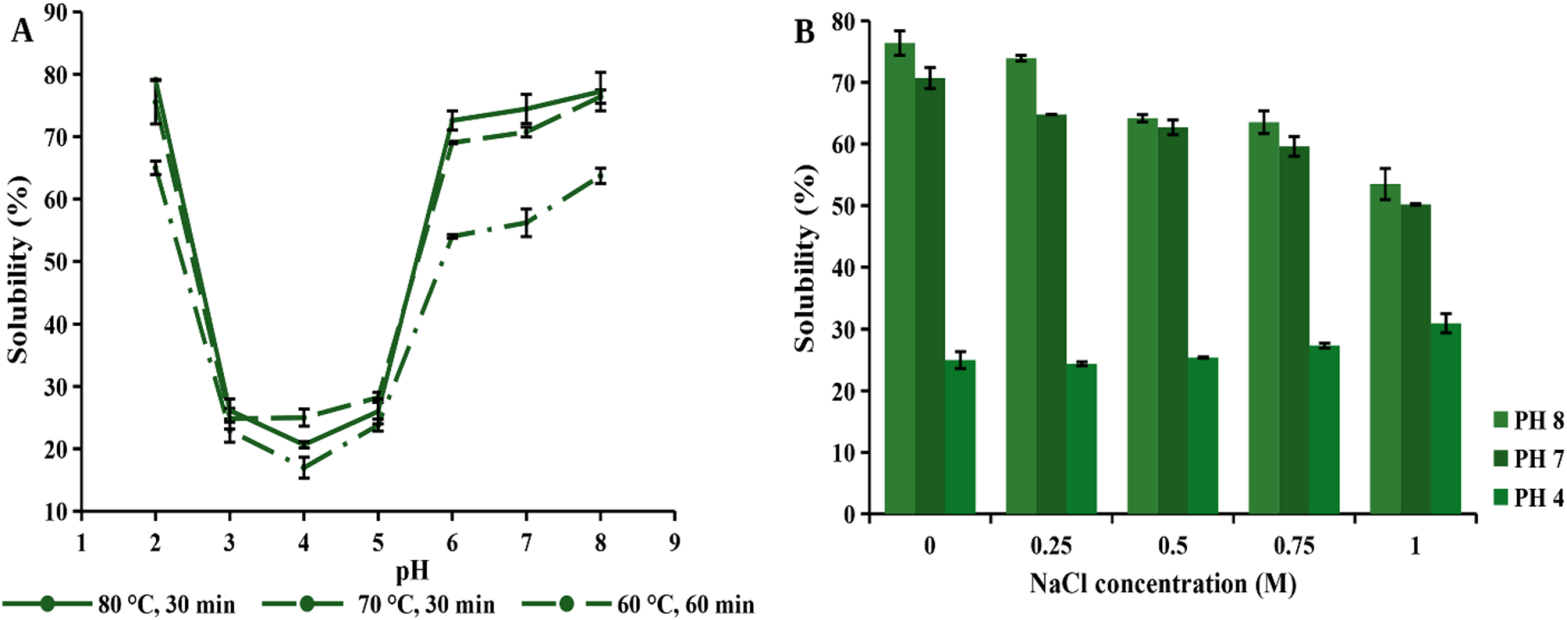
**(A)** Effect of pH on the solubility of green proteins modified by pH-shift treatment. **(B)** Salt effect on protein solubility under conditions of 70 °C for 30 min

The solubility of the obtained modified green protein powder under selected treatment conditions (80 °C for 30 min, 70 °C for 30 min, and 60 °C for 60 min) was additionally examined across a broad pH range and compared, as shown in Fig. 8(A). As the previous results suggested (pH-shift and SDS-PAGE), a final pH of 8 was chosen for further analysis.

The previously broad low solubility region (untreated sample) has now narrowed significantly, becoming concentrated just around the isoelectric point. A quite similar protein solubility trend for modified protein by pH-shift treatment compared to the control was observed by (Alavi et al. 2021). It should be noted that after lyophilization, a slightly lower solubility was obtained compared to the previously analyzed values at pH 7 and 8, likely due to the drying effect on the protein itself. As shown, protein solubility was significantly improved, displaying a similar trend across all treatment conditions. An almost linear increase in protein solubility from pH 6 was detected, along with remarkably high protein solubility in an extremely acidic environment. However, protein solubility in the pH range of 3–5 remained relatively low, indicating that pH-shift treatment could only improve protein solubility around the isoelectric point to a certain extent.

As expected, the results showed that higher temperature had a favorable effect on protein solubility. A considerable increase was observed when the temperature was raised from 60 °C to 70 °C, with a 20.55% higher protein solubility detected at neutral pH. On the other hand, a further increase in temperature did not cause a significant change in protein solubility, which could be associated with protein denaturation, as above results suggested (SDS-PAGE smearing effect). Therefore, to reduce energy consumption, a temperature of 70 °C, which resulted in only slightly lower solubility, was selected for further evaluation.

### Effect of salt on the solubility of modified green proteins

The next step in protein functionality evaluation is the analysis of the salt effect on protein solubility. It is well known that salt can either enhance or impair protein solubility, depending on the concentration, as salt ions and proteins compete for available water molecules. The positive impact of salt is the salting-in effect, while the negative impact is the salting-out effect (Zayas 1997). The impact of salt concentration (NaCl) on protein solubility (treatment conditions 70 °C for 30 min) at neutral, mild alkaline and acidic pH is shown in Fig. 8(B).

It can be concluded that the relationship between protein solubility and salt concentration is rather complex, as it also depends on the pH of the medium. A similar trend was observed at pH 7 and pH 8. The results showed that protein solubility remained relatively stable at a 0.25 M salt concentration. However, a slight decrease in solubility was noted as the salt concentration increased, with a significant decline observed at 1 M, likely due to the salting-out effect (Tanger et al. 2021). Interestingly, the effect of salt on protein solubility at pH 4 exhibited a different trend. As shown, protein solubility remained stable across all salt concentrations, but it was still quite low. A remarkable increase of 23.24% was observed at 1 M salt concentration, probably as a result of the salting-in effect, demonstrating a significant improvement in protein solubility at the isoelectric point.

The results show quite stable solubility, up to 1 M NaCl concentration, of the pH-shift treated green proteins at pH 7 and pH 8, which provides potential applications in various products with a wide range of salt concentrations.

## FTIR- spectroscopy and amide I bend deconvolution in the structural analysis of green proteins

Characteristic protein bands, amide I and amide II, arise from amide bond vibrations. As amide bonds define the secondary protein structure through hydrogen bonding, protein structural modifications can be observed in the FTIR-spectrum (Gallagher 2009). The full FTIR spectrum of pH-shifted (70 °C for 30 min) green protein powder compared to the control is shown in the Supplementary material, Figure S2. Significantly lower intensity was detected in the pH-shifted protein bands (amide I, amide II and amide A region) compared to the control. This result indicates that the protein underwent structural modifications, which is expected, as the pH-shift treatment involves the protein unfolding followed by partial refolding, resulting in a rearranged protein structure (Li et al. 2020).

Furthermore, the secondary protein structure can be defined through the deconvolution of the amide I band, in the range of 1600 to 1700 cm⁻¹, as presented in Figure S3 (supplementary material). The peaks obtained from spectral deconvolution were characterized as follows: wavenumber ranges of 1610–1642, 1643–1650, 1651–1659, and 1660–1699 cm⁻¹ (Gallagher 2009) correspond to β-sheet, random coils, α-helix and β-turn respectively, as shown in Table 4. The results revealed that the pH-shift treatment of green protein induced significant structural changes compared to the control sample, as the most of the secondary structure elements were rearranged.

It is interesting that other plant proteins did not exhibit significant structural changes following pH-shift treatment (Yang et al. 2020; Zhang et al. 2020). This could be explained by the distinct structural composition of the protein, as well as the treatment conditions. On the other hand, the combined effect of temperature and pH is quite complex, as explained in the study Ngui et al. 2021; where major structural changes were observed.

The analysis of the green protein’s structural changes showed that the content of random coil significantly decreased after pH-shift treatment. The high content of this disordered structural conformation (49.9%) can be attributed to the isolation method itself, as thermal coagulation denatures the green proteins, altering their native structure. In contrast, a significant increase in other structural elements was observed, primarily in α-helix content (34.82%).

In order to more precisely evaluate the structural alterations of green proteins, the sample was purified due to potential absorption band overlap in the β-sheet region (Gallagher 2009; Kostryukov et al. 2023; Akhtar et al. 2016) as carbohydrates constituted up to 18.39% (see Table 1) of the green protein powder. Additionally, polysaccharides such as cellulose, hemicellulose, and pectin may interact with proteins through hydrogen bonding or electrostatic interactions, potentially influencing protein folding and stabilization patterns (Rosenberg et al. 2024; Guerrero et al. 2014). The results showed that purified pH-shift sample demonstrated an even more pronounced increase in α-helix content (from 34.82% to 55.57%), indicating a higher degree of structural order and stability following purification. A different trend was observed for β-sheet content, with a decrease from 51.06% to 37.33%. This reduction may be attributed to decreased carbohydrate-protein interactions and a diminished presence of non-protein impurities. Furthermore, as demonstrated in the supplementary material (Figure S3), the presence of well-defined, sharp bands, typically associated with proteins, suggests that spectral overlap of carbohydrates is unlikely to have a significant influence. However, it should be considered that some inevitable protein loss occurred during purification, possibly due to the removal of loosely associated protein complexes, which may have contributed to an altered protein profile. A significant increase in α-helix content accompanied by a reduction in β-sheet structures suggests that the purification process facilitates the correct refolding of the protein. Moreover, the return of β-turn content to levels characteristic of the native state indicates restored structural stability. This structural alteration aligns with previous studies on plant-derived proteins modified through pH-shift treatment (Chen et al. 2024; Jiang et al. 2009).

The changes in the secondary structure of green proteins, predominantly *α*-helix content, were accompanied by an extensive increase in solubility, as the results show. However, to establish the close relationship between the structural changes of green proteins and their solubility, as well as other functional properties, further research and a more detailed analysis of this phenomenon are needed.

**Table 4:** Composition of the secondary structures of green proteins

Secondary structure	Band assignment in green proteins from pumpkin leaves Native pH-shift Purified pH-shift		
*β*-sheet	42.56%	51.06%	37.33%
Random coil	49.90%	n.d.	n.d.
α-helix	n.d. %	34.82%	55.57%
*β*-turn	7.54%	14.13%	7.11%

* Native, green proteins isolated from pumpkin leaf biomass through thermal coagulation process

** pH-shift, conditions (70 °C for 30 min)

*** purified pH-shift (see purification method at Materials and Methods, Fourier-transform infrared spectroscopy section) under same treatment conditions (70 °C for 30 min)

### Microscopic images of green proteins

**Fig. 9:**
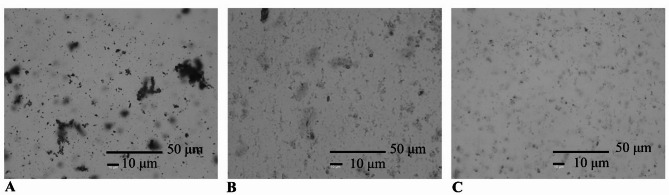
Coomassie Blue Brilliant stained microscopic images of protein samples. **(A)** native (control), **(B)** Protein modified by pH-shift treatment, and **(C)** supernatant of the treated protein under conditions (70 °C for 30 min), dark blue shapes represent proteins. Microscopic images were captured at 400× magnification; scale bars correspond to 10 μm and 50 μm, as labeled

In order to visualize the differences between control and treated protein samples (70 °C for 30 min), Coomassie blue brilliant staining was used, presented in Fig. 9. Before staining, visibility was minimal, particularly in the treated protein sample (result not shown). After addition of the dye, aggregation during application was observed, becoming increasingly pronounced over time. That said, it can be concluded that the dark blue shapes observed were proteins, similar results were also reported by Mustafa et al. (2019). As expected, the native sample displayed significantly more prominent aggregates, which were clearly visible. Compared to the control, the samples modified by pH-shift treatment showed disruption of the tightly bonded structure, resulting in a size reduction. This result was aligned with the detected reduction in average particle size and a more uniform particle size distribution.

## Conclusion

The results highlight the high application potential of green proteins derived from pumpkin leaves, as an underutilized byproduct, in various food applications. The resulting crude green protein powder exhibited a satisfactory protein content typical of protein concentrates, an excellent amino acid profile exceeding FAO/WHO reference values, and confirmed antioxidant properties through ABTS radical scavenging activity and ferrous ion chelation.

The functional properties of green proteins were further improved through an optimized pH-shift and heat treatment. Significant improvements in solubility were achieved in a more alkaline environment and 70 °C for 30 min, particularly at a final pH of 8, leading to a shorter extraction time, reduced particle size, and a more uniform particle size distribution. Furthermore, the modified green protein exhibited excellent stability in NaCl solutions up to 1 M. These changes were accompanied by structural rearrangements, including the formation of new protein interactions (particularly around 25 kDa, likely via disulfide bonds) and significant secondary structural changes confirmed by FTIR, primarily the rearrangement of random coils to α-helices. An increased α-helix content is frequently correlated with improved solubility and enhanced functional properties, such as foaming and emulsifying capacities, which are particularly beneficial in food applications.

Considering all factors, this study demonstrates that green proteins hold great potential as an alternative protein source, enabling the complete utilization of leaf biomass. Furthermore, the pH-shift modified green proteins, with their improved solubility and structural properties, open up numerous possibilities for application in food systems. This remains an active research area, with future efforts directed towards the utilization of modified green proteins. One promising direction is the formulation of modified green protein-stabilized emulsions, as supported by existing literature. Furthermore, an interesting approach involves incorporating active ingredients by employing these proteins as nanocarriers for bioactive compounds - an area of growing interest in recent scientific work. Overall, this approach positions green protein extraction and functionalization as a valuable strategy to create new value chains- from oil producers and green biorefineries to end food consumers.

## Supplementary Information

Below is the link to the electronic supplementary material.

**Supplementary Material 1**: The following supporting information can be downloaded at: … **Figure S1**: Electrophoresis profile of modified green protein samples after pH-shift treatment, without centrifugation. Under reducing (at 70 °C for 30 min and at 80 °C for 30 min, respectively) and non-reducing conditions (at 70 °C for 30 min and at 80 °C for 30 min, respectively). **Figure S2**: FTIR spectra of the green proteins isolated from pumpkin leaf biomass. The solid line represents the sample of modified green proteins under optimal conditions (70 °C for 30 min), while the dashed line represents the control (native) sample. **Figure S3**: FTIR spectra of the amide I band of green proteins for deconvolution. The control is represented by a dark line, pH-shifted sample by a light line and purified pH-shift sample by medium dark line. **Figure S4**: Antioxidative activity of crude green protein over time, measured at different protein concentrations. (A) ABTS radical scavenging assay, (B) Fe^2+^ ion chelation assay.

## Data Availability

All data generated or analyzed during this study are included in this published article [and its supplementary information files]. And raw material data will be made available on reasonable request.
