# Supplementary material for: Exploring green proteins from pumpkin leaf biomass: assessing their potential as a novel alternative protein source and functional alterations via pH-Shift treatment
Source: Bioresour Bioprocess. 2025 Sep 23;12(1):102. doi: 10.1186/s40643-025-00945-x (PMC12457265; doi:10.1186/s40643-025-00945-x)
Supplement: Supplementary file 1 — Supplementary Material 1: The following supporting information can be downloaded at: … Figure S1: Electrophoresis profile of modified green protein samples after pH-shift treatment, without centrifugation. Under reducing (at 70 °C for 30 min and at 80 °C for 30 min, respectively) and non-reducing conditions (at 70 °C for 30 min and at 80 °C for 30 min, respectively). Figure S2: FTIR spectra of the green proteins isolated from pumpkin leaf biomass. The solid line represents the sample of modified green proteins under optimal conditions (70 °C for 30 min), while the dashed line represents the control (native) sample. Figure S3: FTIR spectra of the amide I band of green proteins for deconvolution. The control is represented by a dark line, pH-shifted sample by a light line and purified pH-shift sample by medium dark line. Figure S4: Antioxidative activity of crude green protein over time, measured at different protein concentrations. (A) ABTS radical scavenging assay, (B) Fe2+ ion chelation assay. [file 40643_2025_945_MOESM1_ESM.docx]

**SUPPLEMENTARY MATERIAL**

**Exploring Green Proteins from Pumpkin Leaf Biomass: Assessing Their Potential as a Novel Alternative Protein Source and Functional Alterations via pH-Shift Treatment**

**Marija Korićanac ^1^, Jelena Mijalković ^1^, Predrag Petrović ^2^, Neda Pavlović ^2^ and Zorica Knežević-Jugović ^1^**

**^1^ University of Belgrade, Faculty of Technology and Metallurgy, Department of Biochemical Engineering and Biotechnology, Karnegijeva 4, 11000 Belgrade, Serbia;** [**mkoricanac@tmf.bg.ac.rs**](mailto:mkoricanac@tmf.bg.ac.rs) **;** [**jjovanovic@tmf.bg.ac.rs**](mailto:jjovanovic@tmf.bg.ac.rs)

**^2^ Innovation Centre of the Faculty of Technology and Metallurgy Ltd., Karnegijeva 4, 11000 Belgrade, Serbia;** [**ppetrovic@tmf.bg.ac.rs**](mailto:ppetrovic@tmf.bg.ac.rs) **;** [**nedanikolic@tmf.bg.ac.rs**](mailto:nedanikolic@tmf.bg.ac.rs)

***Correspondence:** [**zknez@tmf.bg.ac.rs**](mailto:zknez@tmf.bg.ac.rs) **; phone.: +381-11-3303776**


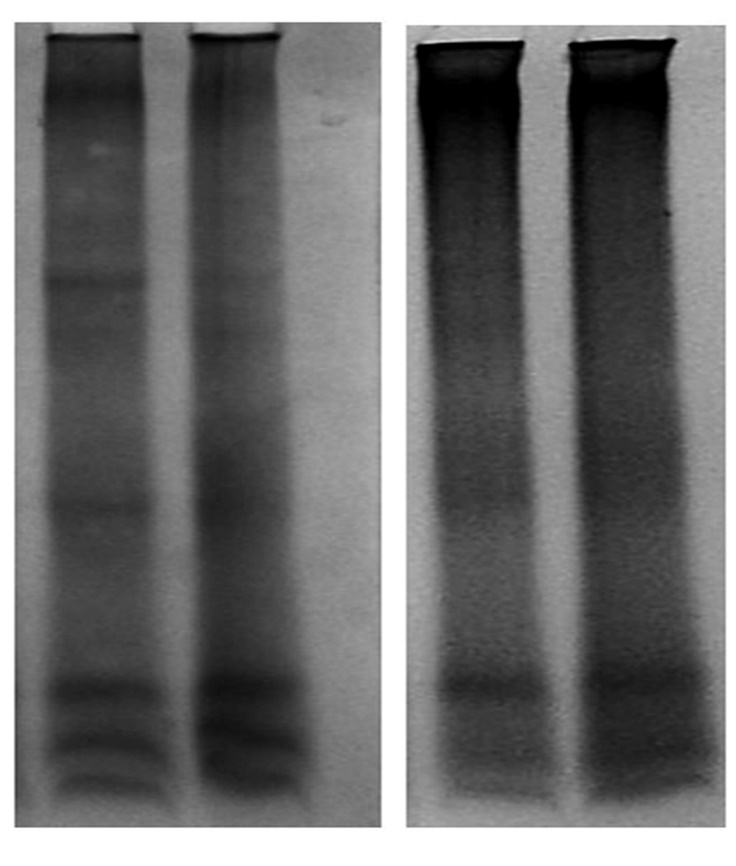


**Figure S1:** Electrophoresis profile of modified green protein samples after pH-shift treatment, without centrifugation. Under reducing (at 70°C for 30 minutes and at 80°C for 30 minutes, respectively) and non-reducing conditions (at 70°C for 30 minutes and at 80°C for 30 minutes, respectively)

**
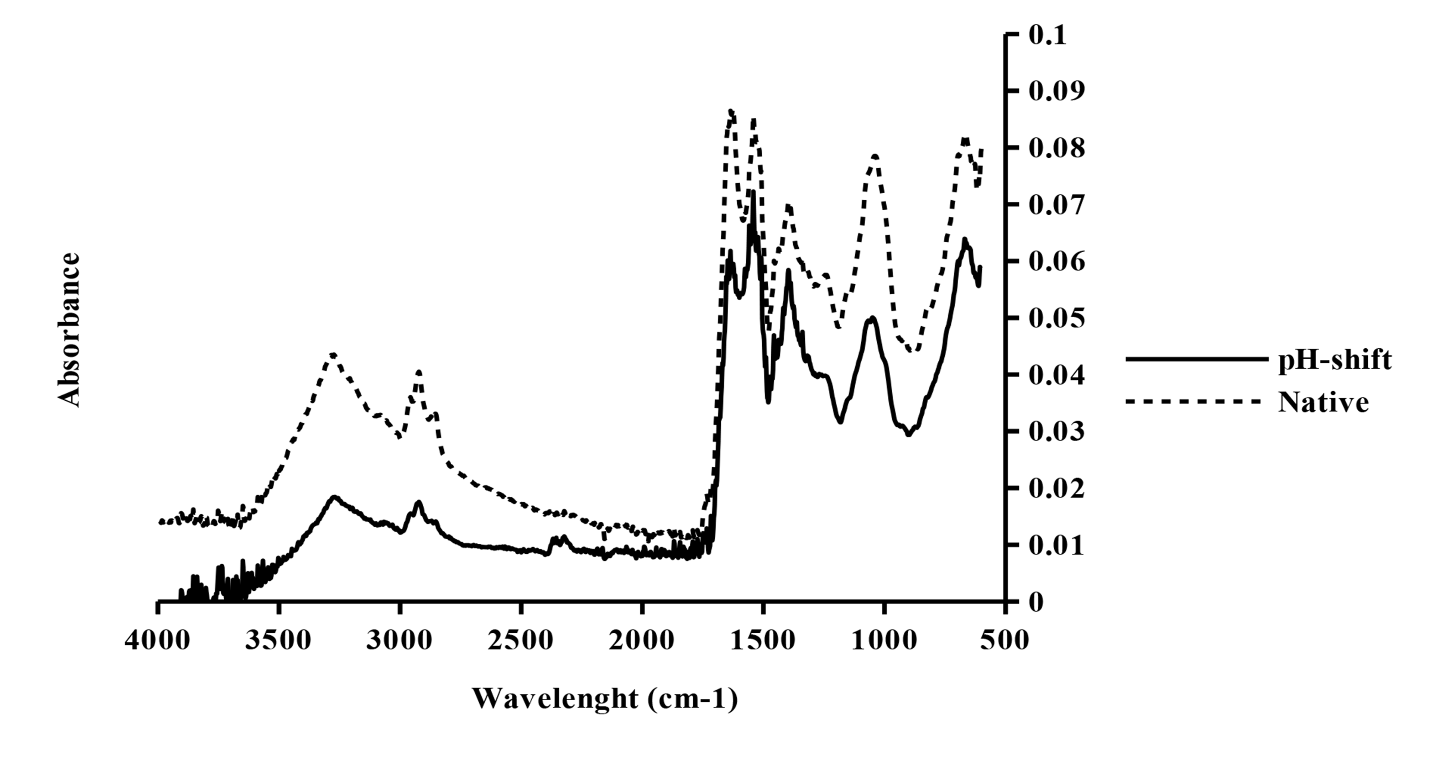
**

**Figure S2****:** FTIR spectra of the green proteins isolated from pumpkin leaf biomass. The solid line represents the sample of modified green proteins under optimal conditions (70 ºC for 30 min), while the dashed line represents the control (native) sample.


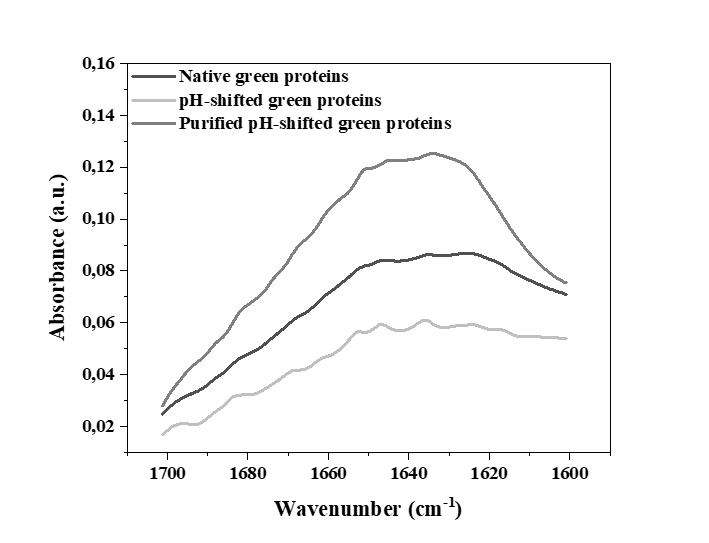


**Figure S3:** FTIR spectra of the amide I band of green proteins for deconvolution. The control is represented by a dark line, pH-shifted sample by a light line and purified pH-shift sample by medium dark line.


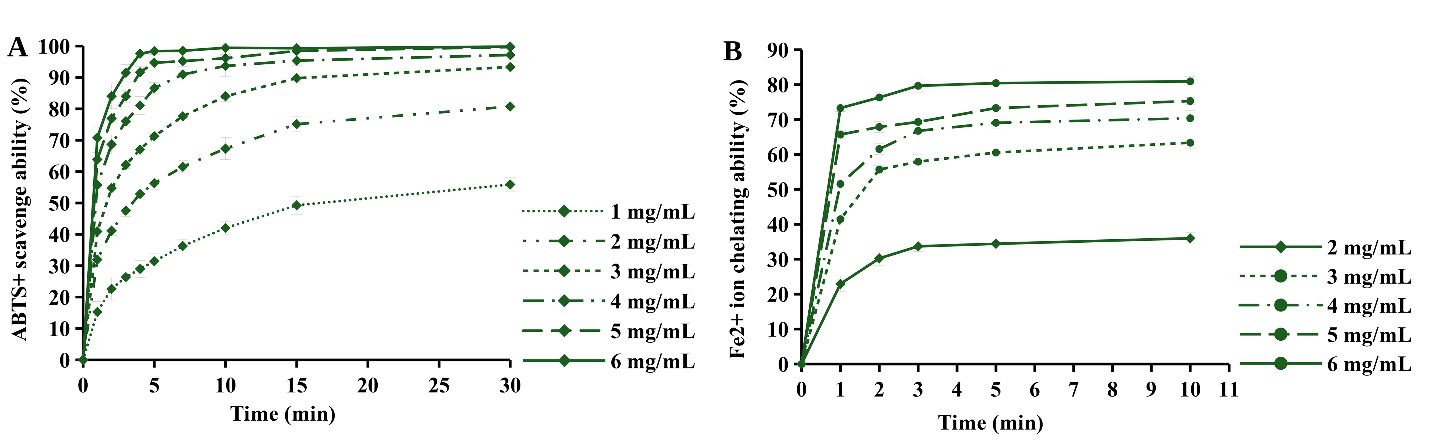


**Figure S4:** Antioxidative activity of crude green protein over time, measured at different protein concentrations. A) ABTS radical scavenging assay, B) Fe^2+^ ion chelation assay.
